# Metagenome-assembled genomes recovered from the datasets of a high-altitude Himalayan hot spring Khirganga, Himachal Pradesh, India

**DOI:** 10.1016/j.dib.2021.107551

**Published:** 2021-11-06

**Authors:** Shekhar Nagar, Chandni Talwar, Meghali Bharti, Sheetal Yadav, Sneha Siwach, Ram Krishan Negi

**Affiliations:** Department of Zoology, University of Delhi, Delhi 110007, India

**Keywords:** Hot spring, Himalayas, Microbes, Metagenome-assembled genomes, Khirganga

## Abstract

Khirganga, a pristine hot spring that lies in the Parvati Valley within the Northern Himalayas characterised with unique white colour microbial mat and divine water with healing abilities. Here, we report 41 metagenome-assembled genomes (MAGs) reconstructed from the microbial mat, sediment and water samples of hot spring passed through Genome Standards Consortium (GSC) and Minimum Information of Metagenome-assembled Genome (MIMAG).

## Specifications Table

**Table:** 

Subject	Environmental Science
Specific subject area	Environmental Genomics and Metagenomics
Type of data	Table
How the data was acquired	Shotgun DNA sequencing using Illumina HiSeq 2500
Data format	Analysed Filtered
Description of data collection	The environmental samples were collected from a hot spring. DNA was extracted for metagenomes sequencing. Genome sequences of 39 Bacteria and 2 Archaea were reconstructed from the metagenome datasets.
Data source location	• City/Town/Region: Khirganga/Kullu/Himachal Pradesh,• Country: India• Latitude and Longitude: 31°59*′*34*″* N, 77°30*′*35*″* E
Data accessibility	• Data is submitted to NCBI GenBank in the public repository. URL to data https://www.ncbi.nlm.nih.gov/bioproject/?term=PRJNA673998; other accessions included with the paper.

## Value of the Data

• This data provides information about genetic potential of bacterial and archaeal candidates in mesothermic hot spring.
• The thermozymes of the metagenome-assembled genomes will be beneficial for sewage treatments and biotechnological processes.
• Data is applicable for comparative genomic studies of 41 different candidates of prokaryotes.
• Data will help to explore the functional potential and inter-habitat interactions of hot spring ecosystem.

## 1 Data Description

A total of 41 Metagenome-Assembled Genomes (MAGs) deconvoluted from water (n=24), microbial mat (n=12) and sediment samples (n=5) which are further described in Table 1. The completeness, contamination and strain heterogeneity of individual MAGs were assessed in CheckM v1.1.3 [1] where 11 high-quality MAGs were produced with >90% completeness and <3.64% contamination and 30 medium quality drafts were estimated at >50% completion and <5.62% contamination qualified the Genomic Standards Consortium (GSC) guidelines [2]. The taxonomic placements of these MAGs were identified with Genome Taxonomy Database GTDB-Tk [3,4] which assign domain to the bins using 120 bacterial and archaeal specific marker genes. The closely related strains were selected using average nucleotide identity (ANI) with 24,706 microbial genomes present in the database using FastANI [5]. The 41 genomes reported here are distributed in the following placements *Alphaproteobacteria* (n=8), *Gammaproteobacteria* (n= 5), *Deltaproteobacteria* (n= 5), *Betaproteobacteria* (n=3), *Bacteroidetes* (n=2), *Cyanobacteria* (n= 2), *Chloroflexi* (n=3), *Defferebacteres* (n=3), *Archaea* (n=2), *Armatomonadetes* (n=2), *Verrucomicrobia, Nitrospirae, Spirochaetes, Actinobacteria, Candidatus Hydrogenedens* and *Ignavibacteria*. The genome sizes, GC content, coding sequences and accession number are summarized in Table 1. These metagenome-assembled genomes will be beneficial for industrial and biotechnological procedures and being mesothermic, the mining will be feasible and their plasmid-host expression would be possible in laboratory [6].

**Table 1:** General features of metagenome-assembled genomes from Khirganga hot spring microbial mat, sediment and water samples.

MAG no.	Taxonomic classification	Sample name*	Genome size (bp)	GC content (%)	No. of Contigs	N_50_(bp)	Coding sequences (CDS)	CMP^a^	CNT^a^	Biosample ID	Accession no.
W46	*Asticcacaulis* sp.	W1	3,436,706	61.4	320	10000	3107	97.98	1.82	SAMN16863472	AIWNX000000000
W26	*Zymomonas* sp.	W1	1,622,650	45.9	184	10000	1938	97.87	1.81	SAMN16863488	JAIWON000000000
W122	*Pararheinheimera* sp.	W1	3,921,803	46.7	379	10000	3678	97.81	1.85	SAMN16863483	JAIWOI000000000
M1	*Buttiauxella noackiae*	M2	4,222,606	49.6	405	10000	3985	96.98	1.73	SAMN16861147	JAIWNL000000000
M59	*Methanospirillum hungatei*	M2	3,005,883	46.2	300	10646	3372	96.79	0.33	SAMN16861154	JAIWNS000000000
W57	*Flavobacterium* sp.	W1	2,527,226	32.5	238	10000	2372	96.2	1.33	SAMN16863480	JAIWOF000000000
M101	*Shewanella putrefaciens*	M2	4,049,599	44.3	383	10000	3767	96	1.46	SAMN16861155	JAIWNT000000000
W28	*Calditerrivibrio* sp.	W1	1,787,395	33.9	171	10,125	1781	93.51	1.75	SAMN16863475	JAIWOA000000000
W65	*Magnetospirillum* sp.	W1	3,533,207	64.6	328	10000	3468	92.74	2.28	SAMN16863481	JAIWOG000000000
W63	*Nitrospira* sp.	W1	2,262,816	58.5	221	10000	2394	91.76	3.64	SAMN16863482	JAIWOH000000000
W18	*Desulfovibrio* sp.	W1	2,958,331	64.3	340	10495	2820	91.14	1.78	SAMN16863479	JAIWOE000000000
M98	*Candidatus Hydrogenedens* sp.	M2	3,130,928	36.9	295	10000	2608	89.82	5.62	SAMN16861152	JAIWNQ000000000
M47	*Desulfomonile* sp.	M2	3,481,803	56.9	317	10000	3592	88.6	0.16	SAMN16861150	JAIWNO000000000
W96	*Thiobacillus* sp.	W1	2,032,255	64.2	192	10000	2083	87.1	0.24	SAMN16863486	JAIWOL000000000
W21	*Thiobacillus* sp.	W1	2,139,896	65.6	229	10702	2186	86.83	2.96	SAMN17050110	JAIWOS000000000
W247	*Armatimonadetes bacterium*	W2	2,598,262	68.5	398	7723	2393	84.72	0.93	SAMN17050106	JAIWOO000000000
M29	*Calditerrivibrio* sp.	M2	1,363,117	34.1	182	9986	1382	84.05	1.32	SAMN16861148	JAIWNM000000000
M74	*Treponema* sp.	M2	2,660,714	49.2	251	10902	2402	83.62	0	SAMN16861156	JAIWNU000000000
W35	*Dechloromonas hydrophilus*	W1	2,286,530	63.4	213	10000	2199	83.12	1.32	SAMN16863478	JAIWOD000000000
W223	*Desulfovibrio* sp.	W2	2,702,232	64.4	442	6860	2612	77.27	1.18	SAMN17050108	JAIWOQ000000000
S239	*Chloroflexi bacterium*	S2	2,873,784	56	527	6258	2899	74.19	1.97	SAMN21434986	JAIUYD000000000
W1106	*Armatimonadetes bacterium*	W1	2,476,173	68.4	372	8290	2299	72.78	2.31	SAMN16863471	JAIWNW000000000
W128	*Yonghaparkia* sp.	W1	1,622,650	70.6	264	7100	1535	72.16	0.51	SAMN16863487	JAIWOM000000000
W148	*Cyanobacteria bacterium*	W1	1,602,259	48	278	6352	1735	71.97	0.31	SAMN16863477	JAIWOC000000000
W1111	*Prosthecobacter* sp.	W1	2,946,816	60.4	560	5688	2698	71.92	0.68	SAMN16863484	JAIWOJ000000000
W166	*Rhizobiales bacterium*	W1	2,057,070	67.8	370	6243	1999	71.09	0.18	SAMN16863485	JAIWOK000000000
W191	*Caenispirillum* sp.	W1	2,568,406	69.7	448	6320	2467	70.87	4.62	SAMN16863474	JAIWNZ000000000
W211	*Rhizobiales bacterium*	W2	2,002,513	67.8	366	5810	1973	69.65	0	SAMN17050109	JAIWOR000000000
W191	*Calditerrivibrio* sp.	W1	1,274,571	34.0	189	8214	1270	68.19	1.75	SAMN17050107	JAIWOP000000000
W8	*Rhizobium* sp.	W1	3,680,731	63.6	351	10000	3638	67.24	1.72	SAMN16863470	JAIWNV000000000
M45	*Klebsiella quasipneumoniae*	M2	4,482,000	58.7	420	10000	4326	66.88	0	SAMN16861153	JAIWNR000000000
M38	*Anaerolinea* sp.	M2	1,832,793	55.6	168	10000	1608	64.09	0.91	SAMN16861146	JAIWNK000000000
S21	*Desulfarculus* sp.	S2	2,977,614	67.2	556	5885	2776	63.28	0.22	SAMN21434984	JAIUYB000000000
S288	*Coleofasciculus chthonoplastes*	S2	5,568,430	46.9	1069	5602	6841	61.29	1.04	SAMN21434985	JAIUYC000000000
W56	*Chloroflexi bacterium*	W1	1,600,678	56	157	10000	1594	60.12	0.91	SAMN16863476	JAIWOB000000000
W113	*Caenispirillum bisanense*	W1	4,037,982	69.5	730	6150	3901	59.81	1.87	SAMN16863473	JAIWNY000000000
S243	*Candidatus Nitrosotenuis*	S2	837,479	41.9	100	10628	1031	57.93	0.97	SAMN21434982	JAIUXZ000000000
M71	*Desulfarculus* sp.	M2	1,828,643	68.1	167	10000	1752	57.63	0.69	SAMN16861149	JAIWNN000000000
M142	*Klebsiella quasipneumoniae*	M1	2,164,359	58.8	307	8269	2086	56.03	1.72	SAMN16925408	JAIWNJ000000000
M19	*Flavobacterium piscis*	M1	3,123,451	34	445	8528	2853	52.47	3.73	SAMN16861151	JAIWNP000000000
S72	*Ignavibacterium* sp.	S1	1,525,417	34.7	326	4873	1351	50.7	0	SAMN21434983	JAIUYA000000000

⁎ Metagenome samples marked as M1, M2 reconstructed from microbial mat, S1, S2 from sediment and W1, W2 from water;

a The completeness (CMP) and contamination (CNT) were checked by using CheckM v1.1.3 [1].

## 2 Experimental Design, Materials and Methods

### 2.1 Site description and sample collection

Khirganga hot spring is a meso-thermic extreme environment characterised by white colour microbial mat deposited in and around the flowing hot water stream [7]. This spring is a relatively undisturbed natural setting at Kullu district, Himachal Pradesh, India. Khirganga lies at an altitude (2978 m MSL) with the source of water being the mystical Parvati River. Due characteristics of geothermal energy, high altitude and white microbial mat that are found in the Khirganga ground leads to emission of heavy metals and ions make the site more provocative [8,9]. Microbial mat, sediment and water samples were collected in replicates from three different habitats and water was filtered through 0.45 µm filter (Merck Millipore Ltd., Ireland) under sterile conditions and filtrate was processed for DNA extraction.

### 2.2 DNA extraction

Total community DNA from water (W1, W2) and sediment (S1, S2) samples were extracted using PowerMax Soil DNA isolation kit (MoBio Laboratories Inc., Carlsbad, CA, USA) following the manufacturer's instructions. DNA extraction from microbial mat (M1, M2) samples described elsewhere [10].

### 2.3 Sequencing and assembly

Community DNA was sequenced at Beijing Genome Institute (BGI), Hongkong, China at Illumina Hiseq 2000 platform and 2 × 100bp paired-end libraries with insert size of 350bp were generated. Reads with <Q_20_ quality cut-off were discarded using SolexaQA [11]. A total of 110,861,650 - 152,895,302 reads in all six samples were generated which were assembled into 180,849 - 519,194 contigs using IDBA-UD [12] with insertion length 50 bp, min. *k-mer* 31, max. *k-mer* 93 and other default parameters. The metagenome-assembled genomes (MAGs) were reconstructed combining contigs based on tetra-nucleotide frequency and genome abundance probabilities using MetaBAT v2 (Metagenomic Binning with Abundance and Tetranucleotide Frequencies) [13] using the following parameters *minContig* (minimum contig size) =2500 bp, and *minS* (minimum score of edge for binning) =60.

### 2.4 Annotation of genomes

Additional genome functional annotation was performed automatically using the Prokaryotic Genome Annotation Pipeline (PGAP) [14].

## 3 Data Accessibility

The raw sequence data were deposited at the National Centre for Biotechnology Information (NCBI) database under the project number PRJNA673998. The sequences of metagenomes are available with SAMN16657637; SAMN16632777 for microbial mat, SAMN16657991; SAMN16673719 for sediment, and SAMN16683881; SAMN16683882 for water. The sequences of MAGs are available at GenBank under the genome accessions summarized in Table 1.

## Ethics Statement

The work did not involve human subjects, animals, cell lines or endangered species of wild fauna and flora.

## CRediT Author Statement

**Shekhar Nagar:** Conceptualization, Data curation, Writing – original draft, Methodology, Software; **Chandni Talwar:** Conceptualization, Data curation, Writing – original draft; **Meghali Bharti:** Conceptualization, Validation; **Sheetal Yadav:** Validation; **Sneha Siwach:** Validation; **Ram Krishan Negi:** Conceptualization, Validation, Supervision.

## Declaration of Competing Interest

The authors declare that they have no known competing financial interests or personal relationships that could have appeared to influence the work reported in this paper.
